# Radioresistance in brain tumors: Strategies for improved radiotherapy outcomes

**DOI:** 10.1016/j.bas.2024.102912

**Published:** 2024-08-06

**Authors:** Muthu Thiruvengadam

**Affiliations:** Department of Applied Bioscience, College of Life and Environmental Science, Konkuk University, Seoul, 05029, Republic of Korea; Center for Global Health Research, Saveetha Medical College & Hospitals, Saveetha Institute of Medical and Technical Sciences (SIMATS), Thandalam, Chennai, 602 105, Tamil Nadu, India

Radiotherapy remains the cornerstone treatment for brain tumors and provides significant benefits in terms of survival and quality of life. However, radioresistance, whereby tumor cells can withstand the damaging effects of radiation, poses a significant challenge to its efficacy. Recent advances in molecular biology have shed light on the mechanisms underlying radioresistance and have offered new avenues for therapeutic intervention. This letter explores these mechanisms and discusses potential strategies to enhance radiotherapy outcomes in patients with brain tumors. Radioresistance in brain tumors is a multifaceted problem driven by various molecular and cellular processes. Key mechanisms include tumor cells, which often have enhanced capabilities to repair the DNA damage caused by radiation. Ataxia-telangiectasia mutated (ATM) and Rad3-related (ATR) proteins play crucial roles in detecting DNA damage and initiating repair processes (Zhao et al., 2022). Upregulation of these proteins in brain tumors can lead to increased radioresistance. Tumor hypoxia and low oxygen levels within the tumor microenvironment are critical factors. Hypoxic cells are less sensitive to radiation because oxygen is a potent radiosensitizer (Kabakov and Yakimova, 2021). Hypoxia-inducible factors (HIFs) regulate cellular responses to low oxygen levels and promote survival pathways that contribute to radioresistance. Cancer stem cells (CSCs) are a subpopulation of tumor cells that can self-renew and differentiate into various cell types. These cells are inherently more resistant to radiation because of their efficient DNA repair mechanisms and quiescent nature, which makes them less susceptible to treatments that target rapidly dividing cells (Abou-Antoun et al., 2017). Aberrant activation of pathways, such as PI3K/Akt, MAPK, and NF-κB, can promote cell survival, proliferation, and resistance to apoptosis (Li et al., 2023). In brain tumors, these pathways are often dysregulated, contributing to enhanced resistance to radiation therapy.

Given the complexity of radioresistance, a multifaceted approach is essential to improve radiotherapy outcomes. Recent studies identified several promising strategies (Hintelmann et al., 2022). Inhibitors of DNA repair proteins, such as poly ADP ribose polymerase (PARP) inhibitors, have shown potential for sensitizing tumor cells to radiation. These agents enhance the efficacy of radiotherapy by blocking repair of radiation-induced DNA damage. Strategies to alleviate hypoxia include the use of hypoxia-activated prodrugs and hyperbaric oxygen therapy. Additionally, targeting HIFs with small-molecule inhibitors can reduce the adaptive responses of tumor cells to hypoxia, making them more susceptible to radiation. Therapies targeting CSCs are being actively explored. Agents that inhibit key signaling pathways involved in CSC maintenance, such as Notch, Hedgehog, and Wnt, have demonstrated potential in preclinical studies (Takebe et al., 2015). Combining these agents with radiotherapy may help to eradicate resistant CSC populations. Inhibiting signaling drugs targeting the PI3K/Akt, MAPK, and NF-κB pathways are currently being investigated. These inhibitors can potentially suppress survival signals in tumor cells, enhancing their sensitivity to radiation. The integration of immunotherapy with radiotherapy is a promising area of research. Radiation can enhance the immunogenicity of tumor cells, making them more recognizable by the immune system. Immune checkpoint inhibitors, such as PD-1/PD-L1 inhibitors, can further amplify this effect, potentially leading to improved outcomes (Jagodinsky et al., 2020). Combination of radiotherapy with chemotherapy or targeted therapy can provide a synergistic effect. Drugs, such as temozolomide, which are already part of the standard treatment for glioblastoma, can be paired with radiation to enhance its efficacy. Ongoing clinical trials are evaluating various drug combinations to identify the most effective regimens. Technological advancements in radiotherapy delivery, such as intensity-modulated radiotherapy (IMRT) and proton therapy, allow for more precise targeting of tumors while sparing surrounding healthy tissue (Ma et al., 2023). These techniques can deliver higher radiation doses to tumors, potentially overcoming resistance. The use of genomic and molecular profiling to tailor the treatment of individual patients is an emerging trend. Personalized treatment plans can be developed to optimize radiotherapy outcomes by identifying the specific mutations and biomarkers associated with radioresistance.

Recent studies have provided valuable insights into the molecular mechanisms that underlie radioresistance. For instance, research has shown that the inhibition of DNA repair pathways using drugs such as olaparib (a PARP inhibitor) can sensitize glioblastoma cells to radiation (Rosado and Pioli, 2023). Additionally, trials combining hypoxia-modifying agents with radiotherapy have demonstrated promising results in improving local control of brain tumors. The role of CSCs in radioresistance is being actively investigated, and studies have highlighted the potential of targeting CSC-specific pathways to enhance the efficacy of radiotherapy. Furthermore, immunotherapy combinations are being explored in clinical settings, with early phase trials showing encouraging outcomes. In conclusion, overcoming radioresistance in brain tumors requires a comprehensive understanding of the underlying molecular mechanisms and development of innovative strategies. By targeting DNA repair pathways, modulating hypoxia, eliminating CSCs, inhibiting key signaling pathways, integrating immunotherapy, and utilizing advanced radiotherapy techniques, we can enhance the efficacy of radiotherapy and improve the outcomes in patients with brain tumors. Continued research and clinical trials are essential to translate these findings into effective treatments, ultimately providing new hope for patients facing this challenging disease.

## Disclosure statement

No potential conflict of interest was reported by author.

## Declaration of competing interest

The authors declare that they have no known competing financial interests or personal relationships that could have appeared to influence the work reported in this paper.
